# Identification of a small RhoA GTPase inhibitor effective in fission yeast and human cells

**DOI:** 10.1098/rsob.220185

**Published:** 2023-03-01

**Authors:** Jun Morishita, Paul Nurse

**Affiliations:** ^1^ Laboratory of Yeast Genetics and Cell Biology, Rockefeller University, New York, NY 10065, USA; ^2^ The Francis Crick Institute, 1 Midland Road, London NW1 1AT, UK

**Keywords:** Rho GTPases, small molecule, inhibitors, fission yeast, cell signalling, cancer

## Abstract

The Rho GTPase family proteins are key regulators of cytoskeletal dynamics. Deregulated activity of Rho GTPases is associated with cancers and neurodegenerative diseases, and their potential as drug targets has long been recognized. Using an economically effective drug screening workflow in fission yeast and human cells, we have identified a Rho GTPase inhibitor, O1. By a suppressor mutant screen in fission yeast, we find a point mutation in the *rho1* gene that confers resistance to O1. Consistent with the idea that O1 is the direct inhibitor of Rho1, O1 reduced the cellular amount of activated, GTP-bound Rho1 in wild-type cells, but not in the O1-resistant mutant cells, in which the evolutionarily conserved Ala62 residue is mutated to Thr. Similarly, O1 inhibits activity of the human orthologue RhoA GTPase in tissue culture cells. Our studies illustrate the power of yeast phenotypic screens in the identification and characterization of drugs relevant to human cells and have identified a novel GTPase inhibitor for fission yeast and human cells.

## Graphical abstract

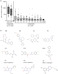

## 1. Introduction

Rho GTPases are a family of highly conserved GTPases that regulate a variety of cell processes involving the actin cytoskeleton [1,2] and are also potential targets for cancer chemotherapies [3–5]. These various processes include adhesion, migration, gene expression, cell division and cell cycle progression [6–9]. Rho GTPases function as molecular switches cycling between an inactive GDP-bound state and an active GTP-bound state. The bound GDP is converted to GTP by guanine nucleotide exchange factors (GEFs), which are critical for Rho activation, localization and stabilization, as well as for interaction with effectors [10]. Once activated, Rho GTPases move to the cell membrane and other cellular compartments to interact with downstream effectors that regulate the cytoskeleton and the dynamics of nuclear and cellular membranes [11]. GTPase signalling is conferred by conformational changes within the switch I and II loops, which dictate binding to effector proteins. The switch I and switch II regions change their conformation when GDP is exchanged for GTP to bring about signalling downstream to effector proteins. Inactivation of GTPase signalling is stimulated by the hydrolysis of Rho-bound GTP to GDP and is promoted by GTPase-activating proteins (GAPs) [12–14]. In addition to these regulators, guanine nucleotide dissociation inhibitors interact with the isoprenylated GDP-bound Rho GTPase to interfere with their translocation from the cytosol to the plasma membrane [15].

The fission yeast *Schizosaccharomyces pombe* encodes six members of the Rho GTPase family: Cdc42, Rho1, Rho2, Rho3, Rho4 and Rho5 [16]. The fission yeast Cdc42 and Rho1 proteins are orthologues of human Cdc42 and RhoA, respectively, and are essential for cell viability [17–19]. Through organizing the actin cytoskeleton, Cdc42 plays a pivotal role in controlling polar cell growth and cytokinesis [17]. Rho1 promotes these events in part through cross-talk with the Cdc42 pathway and plays a role in cell wall integrity by regulating glucan synthesis [16,18,20,21]. Rho2, Rho3, Rho4 and Rho5 are not essential for growth, but they play auxiliary roles for processes related to cell polarity, integrity and division [22–30]. Rho1 is positively regulated by at least three GEFs: Rgf1, Rgf2 and Rgf3 [31–37]. Rgf1 and Rgf2 control the formation of the cell wall, while Rgf3 is involved in cytokinesis [36,37].

During interphase, the growing ends of fission yeast cells contain polarized cables and actin patches. Cortical actin is associated with the deposition of cell wall material at the growing ends [38]. Rod-shaped fission yeast cells grow exclusively from the cell ends. Immediately after cell division, the daughter cells initiate growth in a monopolar manner from the old end, where cortical actin accumulates. In early G2 when the cells reach a certain size, actin starts to accumulate at the new end (the cell end created by cell division) through a process known as new end take-off (NETO), so that the cell switches to a bi-directional growth mode [39,40]. Rho1 localized at the active growth ends is regulated by the GEF Rgf1. Supporting the role of Rgf1 in Rho1-mediated cell integrity, rgf1Δ cells are prone to lyse at one of the poles with a phenotype similar to cells devoid of Rho1. Furthermore, *rgf1*Δ cells show a defect in actin reorganization required for the transition from monopolar to bipolar growth [34]. At mitosis and cell division, actin patches disappear from the poles and the cytokinetic actomyosin ring forms at the cell equator. For these processes, Rho1 couples polarized actin and cell wall biosynthesis through interacting with multiple targets [18,20,21]. Rho1 is localized to active cell growth sites, cell ends and the septum. The depletion of Rho1 activity in growing cells causes the disappearance of polymerized actin, while an increase in Rho1 expression produces larger actin foci that are randomly distributed throughout the cell [20].

Rho GTPases are also critical for growth and division in human cells. Several Rho GTPases are overexpressed in human tumours and abnormal Rho GTPase activities are implicated in a variety of human tumours [41–44]. The upregulation of RhoA is associated with several epithelial human cancer tissues including breast [45,46], testicular [47], liver [48], ovarian [49] and gastric carcinoma [50]. The signalling pathways downstream of Rho GTPases play important roles in cancer cell invasion [51,52], and it has been proposed that the Rho GTPase signalling has an oncogenic role with cancer metastasis [53]. Tumour suppressor functions of RhoA also suggest a context and cell-type specific function for Rho GTPases in cancer. As such, Rho proteins have been explored as potential targets for cancer therapeutics. Small molecule inhibitors targeting RhoGTPase signalling have been developed [51,54–56]. Though molecularly targeted drugs that inhibit Rho GTPases signalling have not yet been widely adopted for clinical use, their potential value as cancer therapeutics continues to drive pharmaceutical research and development.

Here, taking advantage of fission yeast as a model organism to effectively screen drug targets, we report the identification of a novel small molecule inhibitor of Rho GTPase signalling in both fission yeast and human cells. From a diverse library of 10 371 small molecules, we have previously isolated 21 compounds that inhibit normal mitotic progression both in a multiple drug-sensitive fission yeast strain (MDR-sup) [57,58] and HeLa cells [59]. In this paper, we focus on one of them (O1) and show that it is an inhibitor of Rho1 and RhoA in fission yeast and human cells, respectively.

## 2. Results

### 2.1. Identification of non-microtubule-targeting small molecules that perturb mitotic progression in fission yeast

**Figure 1.**
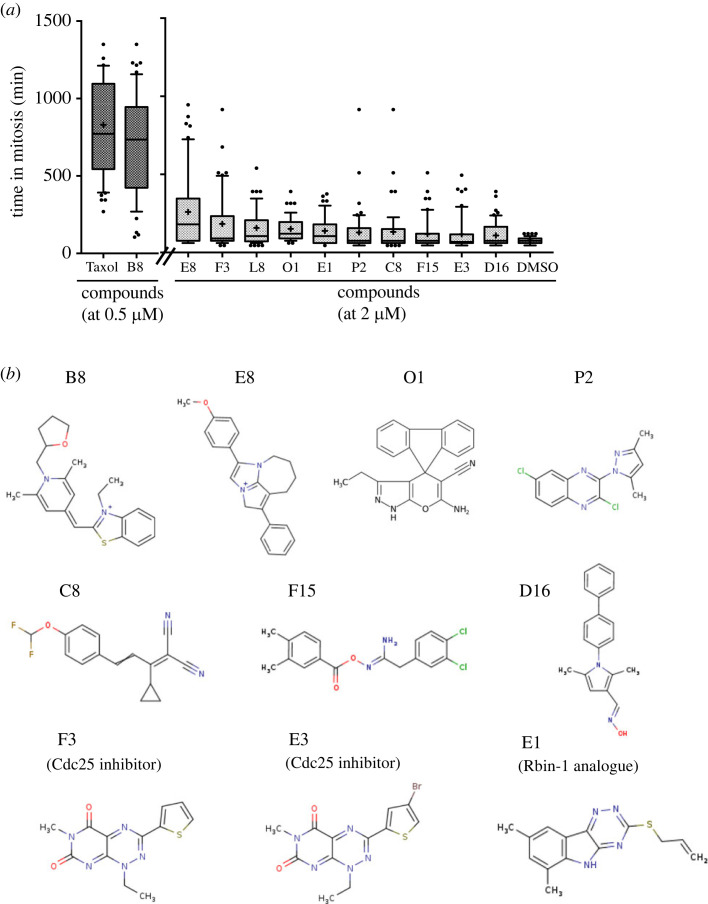
Effect of compounds on mitotic progression in HeLa cells. (*a*) The duration of mitosis was determined by time-lapse microscopy for 24 h in the presence of 0.5 µM (Taxol and B8) or 2 µM (E8, F3, L8, O1, E1, P2, C8, F15, E3, D16) compounds using HeLa cells that were expressing mCherry-tagged histone H2B to mark chromatin and EGFP-tagged alpha tubulin to mark microtubules. The time in mitosis was measured in 200 cells. DMSO-treated cells are shown as the vehicle control. Means are shown as ‘+’ and box represents median value delimited by 10th and 90th percentiles. (*b*) Chemical structures of the 10 mitotic inhibitors screened in this study.

We previously explored a diverse small molecule chemical library to identify mitotic inhibitors, combining screens in fission yeast and HeLa cells, and identified 21 compounds that interfere with mitotic progression in both cell types. Among these compounds, we reported 11 tubulin inhibitors [59]. The remaining 10 compounds showed increases in mitosis duration (figure 1*a*) without any significant inhibitory effects on *in vitro* tubulin polymerization at a concentration of 5 µM (electronic supplementary material, figure S1). In a DMSO control, mitosis duration was 76.5 min, while it was extended by treatment with the following compounds: 0.5 µM B8; 705.9 min, 2 µM E8; 705.9 min, F3; 182.6 min, L8; 157.4 min, O1;151.5 min, E1; 138 min, P2; 128.5 min, C8; 131.9 min, F15; 118.2 min, E3; 116.6 min, D16; 109.0 min. Among them, F3 has been reported as a Cdc25 inhibitor [60], and E3 is a F3 analogue. E1 is an analogue of Rbin-1, which was reported as an inhibitor of Mdn1 required for ribosome biogenesis [61,62]. To test whether Mdn1 is also a target of E1, the sensitivity to E1 was measured using two *mdn1* mutants, *mdn1*^L1113F^ (slightly more sensitive to Rbin-1) and *mdn1^E1187K^* (more resistant to Rbin-1) [61]. E1 exhibited similar sensitivity and resistance responses with Rbin-1, *mdn1*^L1113F^ cells and *mdn1*^E1187K^ cells, respectively (electronic supplementary material, figure S2A). Although E1 was less potent than Rbin-1 in wild-type MDR fission yeast (IC_50_ = 3.8 µM [E1]; 0.89 µM [Rbin-1], electronic supplementary material, figure S2A), E1 might be more specific to Mdn1 proteins than Rbin-1 since *mdn1*^E1187K^ exhibited a more robust growth advantage over wild-type with E1 at the 2–8.3 µM range than with Rbin-1. In HeLa cells, E1 is more effective than Rbin-1 (IC_50_ = 16 µM [E1]; > 50 µM [Rbin-1]; electronic supplementary material, figure S2B), showing delay in prometaphase/metaphase (electronic supplementary material, figure S2C,D).

**Figure 2.**
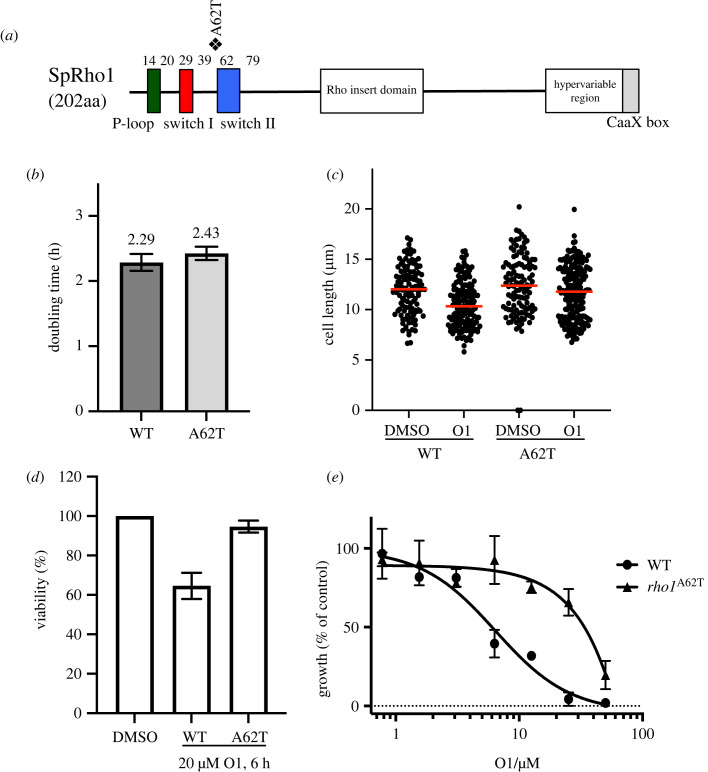
Growth phenotypes of *rho1-A62T* in fission yeast cells. (*a*) Location of the O1 resistance-conferring mutation is indicated. The mutation was in the switch II region which is important for GTP/GDP binding. (*b*) Doubling time of *rho1-A62T* cells and the corresponding isogenic MDR-sup wild-type cells produced from growth curves measuring log phase cells every 2 h at 29°C for 12 h in YES medium. (*c*) Cell length measurements in the presence of O1. Early-log phase cells were incubated in the presence of 20 µM O1 or DMSO control for 6 h at 29°C and cell length was measured. Treating with O1 made cells shorter. Red line, median. *n* = 150 for each sample. (*d*) Percentage of colony-forming units of the *rho1-A62T* strain compared with that of the wild-type strain. Cells incubated with 20 µM O1 for 6 h at 29°C were washed twice and diluted and counted, and the same number of cells was plated on YES medium and incubated for 3 d at 29°C. (*e*) Wild-type (WT) and *rho1-A62T* cells were incubated for 15 h at 29°C with the indicated concentrations of O1. Growth (%) is presented relative to DMSO-treated cells. Each point is the mean value for two independent experiments.

In this study, because of its unique chemical structure, we focused on the compound O1 (figure 1*b*). O1 extended mitotic duration in HeLa cells by arresting them in metaphase (electronic supplementary material, movie) ranging from 126 min (0.5 µM) to 151.5 min (2 µM), compared with a mean mitotic duration of the control DMSO-treated cells at 76.5 min (electronic supplementary material, figure S3). To identify the target of the compound O1, we conducted a suppressor mutant screen in fission yeast. We chemically mutagenized the MDR-sup fission yeast strain and isolated resistant clones that could grow in the presence of 60 µM O1. Genome sequence analysis of a backcrossed resistant mutant revealed a missense mutation in the *rho1^+^* (SPAC1F7.04) gene, resulting in a mutant Rho1 protein with a threonine in place of a highly conserved alanine 62 (A62T, figure 2*a*). The core domain of Rho1 is composed of three conserved motifs: the phosphate-binding loop (P-loop), switch I and switch II (figure 2*a*). These three motifs are critical for the GTPase activity by cooperatively recognizing guanine nucleotides and Mg^2+^ [63]. In the crystal structure of human RhoA (PDB 1A2B), the human Rho1 orthologue, Ala62 is located at the loop of switch II, proximal to the gamma-phosphate of ATP. We generated a *rho1-A62T* mutant by introducing an A62T mutation at the endogenous *rho1^+^* gene by gene replacement. The mutant strain appears to grow normally (figure 2*b*). O1 decreased the average population cell length from 12 µm to 10.3 µm in the wild-type strain after incubation for 6 h at 29°C, whereas no such decrease by O1 was observed in the *rho1-A62T* strain, where average cell size is 12.3 µm in the DMSO control and 11.8 µm in the presence of O1 (figure 2*c*). Cell viability after a 6 h incubation with 20 µM O1 was examined by a colony formation assay. The *rho1-A62T* mutant cells showed little decrease in viability compared with that seen in DMSO control, although viability did decrease more than 30% in wild-type cells after incubating with O1 (figure 2*d*). Cell growth assays in liquid culture also confirmed that the mutant *rho1-A62T* conferred resistance to O1 (figure 2*e*), but not to cycloheximide, a chemically unrelated inhibitor (electronic supplementary material, figure S4). These results suggest that Rho1 is the likely target of O1 in fission yeast.

### 2.2. O1 perturbs actin organization in fission yeast

**Figure 3.**
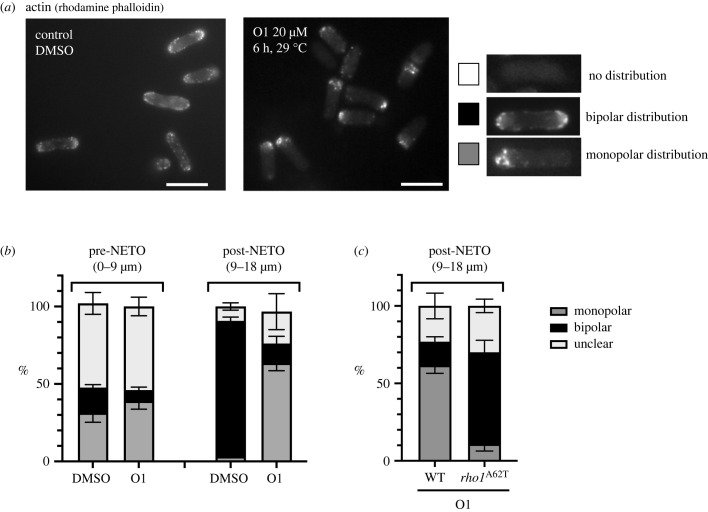
Actin organization was perturbed in the presence of O1. (*a*) Early-log phase wild-type fission yeast cells grown in YES liquid medium at 29°C in the absence (left) or presence (right) of 20 µM O1 were collected after 6 h and actin organization visualized with rhodamine- conjugated phalloidin. bar, 10 µm. (*b*) The graphic represents the percentages of monopolar and bipolar actin growth patterns in preNETO (cell length 0–9 µm) and post-NETO (9–18 µm) cells. (*c*) Actin growth patterns shown for post-NETO in WT and the *rho1-A62T* mutant cells incubated with 20 µM O1, 6 h at 29°C.

To investigate the possible effects of O1 on NETO and cell morphology, the localization of actin was determined using rhodamine-conjugated phalloidin. With a control DMSO treatment, a bipolar actin distribution was only seen in approximately 15% of cells shorter than 9 µm, but was observed in approximately 90% of cells longer than 9 µm, demonstrating that NETO was triggered before reaching a cell length of 9 µm. By contrast, in the presence of O1, approximately 30% of cells longer than 9 µm were bipolar (figure 3*a,b*). In the O1-resistant mutant *rho1-A62T* cells, early NETO was restored, with more than 60% of cells showing a bipolar distribution in cells longer than 9 µm (figure 3*c*).

### 2.3. O1 reduces the amount of cellular GTP-bound Rho1 in fission yeast cells

**Figure 4.**
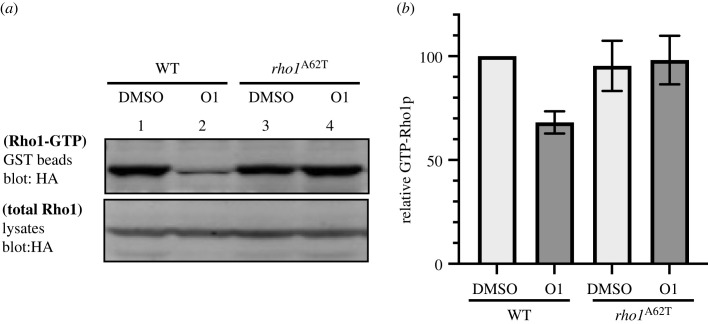
Rho1–GTP activity. (*a*) Extracts carrying HA-rho1 or HA- rho1^A62T^ were precipitated with GST-RBD and blotted with anti-HA (top). Total Rho1 or Rho1^A62T^ protein in cell lysates were also visualized by western blot with anti-HA antibody (bottom). (*b*) Data were quantified from three independent experiments and presented as percentage relative to the wild-type with a DMSO control.

To further investigate the effect of O1 on the Rho1 protein, we examined if O1 affects the *in vivo* amount of the GTP-bound Rho1 protein. Wild-type MDR-sup cells carrying haemagglutinin (HA)-Rho1 were incubated with 20 µM O1 for 6 h, and then the amount of the activated GTP-bound Rho1 was analysed by precipitation with the GST-Rhotekin-binding domain, which only binds to GTP-bound Rho1 but not GDP-bound Rho1. Anti-HA western blots revealed that cell lysates treated with O1 had less GTP-bound Rho1 protein (figure 4*a*, lane 1 versus 2). The whole-cell lysates had similar amounts of total Rho1 protein, suggesting that O1 did not affect the stability of Rho1 (figure 4*a*, lower panel). By contrast, O1 did not affect the amounts of GTP-bound Rho1 protein in *rho1-A62T* cells, while the total amount of Rho1 was not changed by the A62T mutation (figure 4*a*, lane 3 versus 4; figure 4*b*), consistent with the idea that O1 directly destabilizes the Rho1–GTP interaction without affecting protein stability, and that the A62T mutation rescues the interaction.

We next addressed if O1 influences the subcellular localization of the Rho1 and Rho1^A62T^ proteins using constitutively expressed GFP-Rho1 and Rho1^A62T^ in fission yeast MDR-sup wild-type and *rho1-A62T* mutant strains. GFP signals of Rho1 and Rho1^A62T^ proteins were seen in the plasma membrane, and were slightly enriched at the cell tips and at the septum in both strains, as previously described [20,64,65]. Their localizations were not affected by O1 (electronic supplementary material, figure S5). These results suggest that O1 reduces the amount of GTP-bound Rho1 protein without affecting Rho1 protein localization.

### 2.4. The expression of mutant RhoA^A61T^ protein alters the sensitivity to O1 in human cells

**Figure 5.**
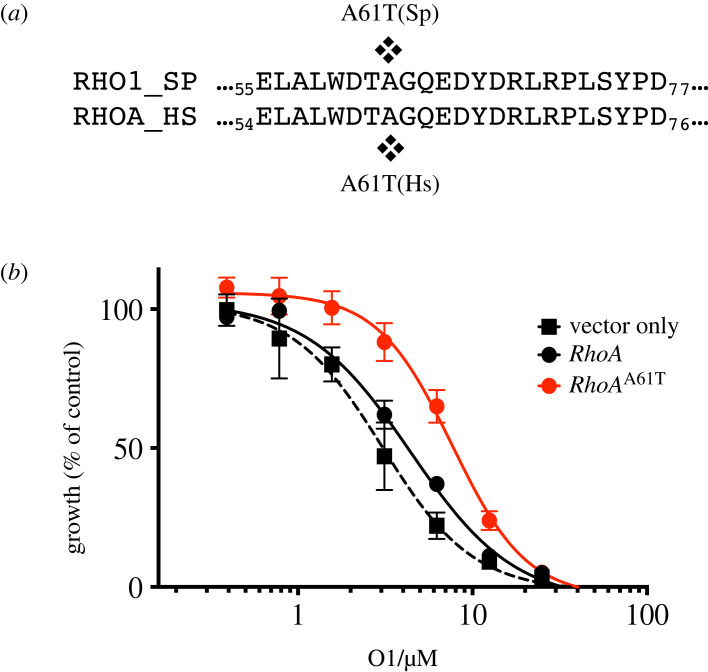
O1 affects the actin cytoskeleton in Flp-In-293 cells. (*a*) The alignment of amino acid sequence in switch II region in fission yeast Rho1 and human RhoA GTPase are shown. (*b*) Dose–response curves in Flp-In 293 cells expressing wild-type RhoA, mutant RhoAA61T and vector only as a control. Cells were incubated with different concentrations of O1 and cell viability was determined after 4 days. Mean ± s.e.m. (*n* = 2 experiments) is shown.

The fission yeast Rho1 GTPase and its orthologue human RhoA GTPase share a high degree of sequence similarity with 89% amino acid identity in the N-terminal half of protein containing the P-loop, switch I and switch II, which form the GTP-binding pocket. The Rho1 Ala62 in fission yeast corresponds to Ala61 in human RhoA (figure 5*a*). To test whether the target of O1 could be RhoA GTPase in human cells, we generated human embryonic kidney Flp-In-293 cell lines stably expressing FLAG-tagged wild-type RhoA, mutant RhoA^A61T^ protein and a vector only control. The IC_50_ of O1 in vector only cells and RhoA-expressing cells were, respectively, 3.3 µM and 4.7 µM, while the cells expressing in mutant RhoA^A61T^, the IC_50_ was 6.9 µM (figure 5*b*). The decreased O1 sensitivity with RhoA^A61T^ suggests that O1 targets RhoGTPase protein in both fission yeast and human cells.

### 2.5. O1 inhibits cellular RhoA activity in tissue culture cells

**Figure 6.**
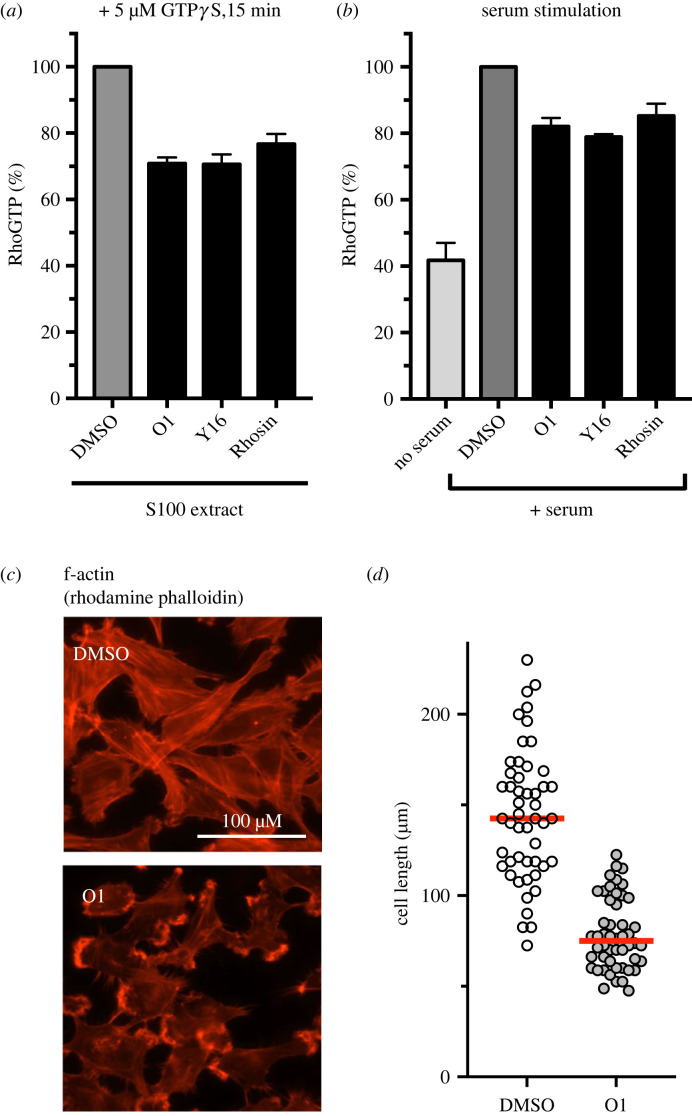
O1 inhibits fully activation of RhoA in HeLa and Swiss 3T3 cells. (*a*) S100 extract from HeLa cells was incubated with 30 µM O1 in the presence of 5 µM GTPγS for 15 min, and RhoA-GTP levels were detected by a G-LISA RhoA activation assay. DMSO result was set at 100%; 30 µM Y16 and 30 µM Rhosin were used as controls, respectively. (*b*) Swiss 3T3 cells were serum-starved for 24 h with or without 30 µM compound and subsequently were stimulated with 10% serum for 15 min. Cell lysates were subject to the G-LISA RhoA activation assay. Compound treatment conditions were normalized to the corresponding DMSO control. (*c*) Effect of O1 on cell stress fibre and focal complex assembly. After serum stimulation, Swiss 3T3 cells were washed three times and then fixed and stained with Rhodamine-phalloidin for F-actin. scale bar represents 100 µM. Red lines, median (*n* = 50 each). End-to-end cell lengths were measured and plotted (*n* = 50).

To further examine if O1 effectively inhibits RhoA in human cells, a RhoA activation assay in an S100 extract from HeLa cell was performed using G-LISA RhoA [66]. In this assay, RhoA-GTP, but not RhoA-GDP, can be detected. To trap the cellular RhoA in the activated form in the presence or absence of O1, non-hydrolysable GTP*γ*S was added to HeLa cell extracts and the amount of RhoA-GTP*γ*S was measured. Two RhoA inhibitors that interfere with RhoA-RhoGEF interaction, Rhosin (targeting RhoA) and Y16 (targeting the RhoGEF LARG), were used as controls [67,68]. The IC_50_ of O1 in human cells was comparable to Rhosin and Y16 (IC_50_ = 6.22 µM (O1); 6.33 µM (Rhosin); 4.75 µM (Y16), electronic supplementary material, figure S6). Neither Rhosin nor Y16 exhibited any growth inhibition of fission yeast cells (electronic supplementary material, figure S7). The fraction of GTP*γ*S-bound form of RhoA was reduced by 30% with 30 µM O1, comparable to Rhosin and Y16 (figure 6*a*).

RhoA is known to be activated during serum activation [69]. To examine if O1 interferes with this process, mouse fibroblast (Swiss 3T3) cells were treated with or without 30 µM O1 in serum-free media for 24 h, followed by stimulation with 10% calf serum for 15 min. O1 suppressed serum-induced RhoA-GTP formation was found to be comparable to that seen with Rhosin and Y16 (figure 6*b*). Upon serum stimulation, RhoA plays a critical role in the cell cytoskeleton and cell shape reorganization [6]. Figure 5*c* shows that the addition of O1 strongly inhibited reorganization of the actin cytoskeleton—both stress fibre and focal adhesion complex formation of the cells were reduced, and cells lost their fibroblastic elongated shape to acquire a more rounded morphology. The median end-to-end cell length after serum stimulation was 142.5 µm in a DMSO control and was shortened to 81.2 µM in the presence of 10 µM O1 (figure 6*d*). Given the role of RhoA in actin cytoskeleton and adhesion, these results support the conclusion that O1 is an inhibitor of RhoA in mammalian tissue culture cells.

## 3. Discussion

Natural and synthetic small molecules that target the cell division machinery are useful tools for dissecting the processes of the cell cycle and cell division. Fission yeast is a powerful organism for chemical and genetic screening, and facilitates a rapid economical workflow. For primary drug screening, we used the drug-sensitive MDR-sup fission yeast strain to identify chemical compounds which inhibit cell growth. The subsequent secondary screening in HeLa cells reduced the numbers of drugs, which are also effective in human cells. Cell cycle specific effects of these selected drugs were then monitored by live imaging in HeLa cells to select drugs that inhibit mitotic processes [34,70]. Among those drugs, we identified O1, which, to the best of our knowledge, is the first inhibitor of Rho1 in fission yeast and will be a useful reagent to dissect the cell cycle and other cellular roles involving Rho1.

Several lines of evidence indicate that O1inhibits Rho1 by interfering with stable formation of Rho1–GTP. The mutation (Ala62 in fission yeast, Ala61 in human) located in the switch II region of Rho1/RhoA made cells resistant to O1. In human cells, the highly conserved residue Gly62 in the switch II of RhoA coordinates the γ-phosphate of GTP. Since the resistant mutation Ala61 is located next to Gly62, it is reasonable to suggest that O1 binds to the switch II region and interferes with GTP binding to RhoA or GDP dissociation from RhoA. Alternately, O1 may affect its regulators either by inhibiting GEFs that facilitate GDP dissociation or by activating GAPs that stimulate GTP hydrolysis. In fission yeast, because the deletion of Rgf1 decreases the amount of GTP-bound Rho1, Rgf1 is considered the Rho1-GEF that is responsible for most of the GTP-bound Rho1 in the cells [5,6]. Since another GEF Rgf2 functions redundantly with Rgf1, *rgf1Δ* cells are viable but fail to activate bipolar growth showing monopolar actin distribution after mitosis. These phenotypes resemble the phenotypes observed when cells are treated with O1. If O1 had inhibited Rho1-GEFs (or activated Rho1-GAP) rather than Rho1, its resistant mutation in Rho1 should have increased the cellular amount of Rho1–GTP even in the absence of O1. However, this was not observed in *rho1-A62T* cells. Unlike *rho1-A62T* mutant, which appears to be normal except for its resistance to O1, *rho1* hypomorph mutants are very sick and show many defective cellular phenotypes [20,65,71]. By contrast, fission yeast cells treated with 20 µM O1 showed only mild phenotypes such as a 35% decrease in viability after 6 h. This is in line with our observation that O1 reduces the amount of GTP-bound Rho1 by about 30%. Therefore, it is most likely that O1 binds to the switch II region of Rho1 protein, but not to Rho1^A62T^, which alone does not affect binding to GTP γ-phosphate. Although a *rho1-A62T* mutant did not show any phenotypes in normal growth conditions at 29°C, because Rho1 Ala62 is highly conserved, it is possible that the A62T mutation affects the function and/or stability under different conditions.

Treating HeLa cells with O1 causes mitotic delay with defective spindle formation. This phenotype was not expected since the established role for RhoA in animal cells is in cytokinesis [72] as GTP-bound RhoA increases from anaphase to telophase [73]. Indeed, the depletion of RhoA by siRNA in HeLa cells results in binuclear cells with defects in cytokinesis [74] . Microinjection of C3 transferase into HeLa cells, which inhibits RhoA, RhoB and RhoC also blocks cytokinesis [64]. By contrast, it was reported that treatment of Rhosin to MCF7 cells induces apoptosis but did not cause cell cycle arrest [67]. Another Rho family protein, Cdc42, has been implicated in kinetochore-microtubule attachment [75], and so it is possible that O1 also targets Cdc42. Indeed, introduction of a mutation that confers resistant to fission yeast Rho1 into human RhoA gave only a modest resistance to O1 in HeLa cells. Therefore, although this partial effect may be due to the presence of endogenous RhoA, it is possible that O1 has additional targets such as Cdc42.

There has been interest in developing small molecule inhibitors for the Rho GTPase signalling pathway as potential therapeutic targets [54] because of their roles in cancer initiation, cancer progression and cancer metastasis [53]. Consistent with this idea, studies have demonstrated roles of Rho GTPases in cancer [76]. However, Rho GTPases may have both oncogenic and tumour suppressor functions, suggesting a context and cell-type specific function for Rho GTPases in cancer. Inhibiting a specific Rho GTPase in a specific context by a small molecule may open up the potential of Rho GTPases as therapeutic targets and prognostic tools for cancer patients. As such, it is important to develop small molecule inhibitors targeting Rho GTPases, and to accumulate evidence from studies in appropriate model systems. Based on our success in O1 identification, we suggest that the consecutive phenotypic screening with fission yeast and human cells, followed by effective isolation and characterization of fission yeast-resistant mutants will be an attractive pipeline to identify inhibitors targeting the Rho GTP pathways.

## 4. Materials and methods

### 4.1. Yeast strain, growth conditions and growth assay

Experiments were conducted in yeast extract (YE) medium containing adenine, leucine, uridine and histidine to a final concentration of 0.15 g l^−1^. The *Schizosaccharomyces pombe* SAK933 strain (h90 ade6 leu1 ura4-D18 GFP-atb2 ≪ kanr sid4-mcherry ≪ hygr caf5 :: bsdR pap1Δ pmd1Δ mfs1Δ bfr1Δ dnf2 Δ erg5::ura4+) [58,59] used for screening in this study was grown at 29°C. All experiments were performed in exponential growth. The A62T mutation was introduced into on rho1+ by PCR and the linear DNA used to transform a MDR-sup strain, SAK950 [58] (h + ade6-M216 leu1 ura4-D18 caf5::bsdR pap1Δ pmd1Δ mfs1Δ bfr1Δ dnf2 *Δ* erg5::ura4+) using the kanMX6 selection marker, and construction was confirmed by sequence analysis. To make GFP- and HA-tagged Rho1 strains, pJK148 plasmids containing N-terminally GFP or a HA epitope-tagged Rho1 or Rho1^A62T^ under control of its native promoter and with its terminator (kindly provided by Pilar Pérez) were used to insert HA-Rho1 or Rho1^A62T^ and GFP-Rho1^+^ or Rho1^A62T^ in the *leu1* locus of MDR-sup wild-type or *rho1-A62T* mutant strains. In a growth assay, logarithmically growing cells were diluted to OD = 0.1 and 10 times dilution was used. One millilitre of the cell culture mixed with the compound at several dilution series was incubated for 15 h at 29°C. The optical density was measured to calculate the growth ratio and DMSO control was used for control.

### 4.2. Isolation of O1 resistant mutants

For mutagenesis, the SAK950 strain treated with 1-methyl-3-nitoro-1-nitrosoguanidine in TM buffer (50 mM Tris, 50 mM Maleic acid, 7.5 mM (NH_4_)_2_SO_4_, 0.4 mM MgSO_4_7H_2_O pH6.0) for 15 min, and incubated in YE medium for 3 h, cultured in YE medium containing 60 µM O1 at 29°C for two weeks in a 96-well plate maintained in log phase growth. Two resistant clones were isolated and backcrossed with SAK950 wild-type for eight times. Sequencing of these clones revealed that these clones had a point mutation A62T in the *rho1^+^* gene. To reconstruct the A62T point mutation in *rho1^+^*, a DNA fragment containing the A62T point mutation was transformed into the SAK950 strain. Homologous recombination between the rho1 mutant fragment and the rho1^+^ gene produced a rho1 mutant strain. The replacement of *rho1^+^* gene by the *rho1*-*A62T* mutation was confirmed by sequencing after amplifying the genome segment using PCR.

### 4.3. Mammalian cell lines

All mammalian cell lines were incubated at 37°C in a humidified incubator containing 5% CO_2_ maintained in log phase growth and were routinely monitored for mycoplasma by PCR (Universal Mycoplasma Detection Kit, ATCC). HeLa cell line, Flp-In-293 cells (gifted from Sohail Tavazoie) and Swiss 3T3 cells (gifted from Sohail Tavazoie) were grown in DMEM (Thermo Fisher Scientific) supplemented with 10% TET-tested FBS (Atlanta Biologicals) and penicillin-streptomycin (100 u ml^−1^, GIBCO). To generate a stable cell line, parent 293 cells were plated onto 6-well plates and cultured for 24 h and cells were co-transfected using 2 µl of TransIT-2020 reagent (Mirus) with 0.3 µg of construct and 1.0 µg of pOG44 (Thermo Fisher Scientific). After 24 h, cells were plated to 60 mm dishes and subject to selection with 50 µg ml^−1^ hygromycin B and 10 µg ml^−1^ blasticidin for a month. A RhoA point mutation A61T was introduced through site-directed mutagenesis PCR using Q5 Site-Directed Mutagenesis Kit (New England Biolabs, cat. no. E0554).

### 4.4. Time-lapse microscopy

Live cell imaging was performed in a LCV110U VivaView FL incubator microscope (Olympus) equipped with an X-Cite-exacte illumination source (Excelitas Technologies) and Orca-R2 CCD camera (Hamamatsu Photonics) at the Rockefeller University Bio-Imaging Resource Center. For screening, images were acquired with a 20X objective every 15 min for 24–48 h in the differential interference contrast, m-cherry and GFP channels [59].

### 4.5. *In vitro* tubulin polymerization assay

A Tubulin Polymerization HTS Assay Kit (Cytoskeleton, BK011P) was used according to the manufacturer's instructions. All components were added to a 96-well microtiter plate (Corning Costar, cat. no. 3686), then the tubulin reaction mixture was quickly added to the wells, and tubulin polymerization initiated and monitored every 1 min at 37°C for 1 h by recording fluorescence of excitation wavelength at 340 nm and emission at 450 nm. The tubulin reaction mixture was composed of 80 mM PIPES (pH 6.9). 1 mM MgCl_2_, 1 mM EGTA, 1 mM GTP and 2 mg ml^−1^ of highly purified porcine brain tubulin heterodimer (Cytoskeleton, cat. no. T240) [59].

### 4.6. Actin staining in fission yeast

Filamentous actin was visualized using Rhodamine-phalloidin staining using a modification of the method as described [77] with a fixation time for 5 min and using 3.7% fresh paraformaldehyde (Electron Microscopy Sciences, USA). Rhodamine-phalloidin 488 (Molecular Probes) was added to the permeabilized cells by vortexing in 100 µl 1% Triton X- 100 in PBS for 1 min and washed 3 x with PBS, and incubated with gentle rotation for 50 min at room temperature. The fluorescent images were obtained by using a fluorescence microscope (inverted Olympus IX-70 microscope).

### 4.7. *In vivo* analysis of Rho1 activity

The amount of GTP-bound Rho1 proteins was analysed using a Rho1–GTP pull down assay. Wild-type, *rho1-A62T* mutant MDR-sup strains integrated HA-Rho1 or HA-Rho1^A62T^ in Leu locus were grown in YE medium and 10^7^ early-log phase cells were obtained as described previously [34,71by using 100 µl of lysis buffer (50 mM Tris-HCl pH 7.5 20 mM NaCl 1 mM ETA 0.05% NP-40 10% glycerol 0.1 mM DTT 1 mM PMSF and 1X complete protease inhibitor cocktail Roche 11873580001]). Ten micrograms of Rhotekin-RBD Beads (Cytoskeleton, Inc. #RT02) were used immunoprecipitated the GTP-bound HA-Rho1 or HA-Rho1^A62T^. The extracts were incubated with GST-RBD beads by rotation at 4°C for 2 h. The beads were washed with lysis buffer four times, and samples were then subjected to gel electrophoresis and western blotting with 1 : 2000-diluted anti-HA (12CA5) mAb to detect HA-Rho1 or Rho1^A62T^. For checking the total amount of HA-Rho1 or Rho1^A62T^, 20 µg of whole-cell total protein was used for western blot with anti-HA mAb and detected using the Odyssey Infrared Imaging System (LI-COR Biosciences).

### 4.8. Actin staining in Swiss 3T3 cells

The cells were fixed with 3.7% formaldehyde in PBS for 15 min and permeabilized with 0.1% Triton X-100 for 20 min. The cells were stained with Rhodamine-phalloidin 488 (Molecular Probes) for F-actin and DAPI for DNA. The fluorescent images were obtained by using a fluorescence microscope (Inverted Olympus IX-70 microscope).

### 4.9. G-LISA RhoA activity assay

For the quantitative analysis of active RhoA-GTP levels, assays were performed using G-Lisa RhoA Activation kit (Cytoskeleton, cat. no. BK124.) according to manufacturer's instructions. This assay uses RhoA-GTP-binding proteins linked to the wells of a 96-well plate. Active, GTP-bound RhoA in cell lysates binds to the wells while inactive GDP-bound forms are removed during wash steps. The amount of bound RhoA-GTP was detected by using primary anti-RhoA antibody followed by a secondary antibody conjugated to FRP. The signal was read by measuring at 490 nm using a microplate reader a Synergy Neo2 (Bio Tek).

#### 4.9.1. Rhoa activation by GTP*γ*S

Non-hydrolysable GTP analogue, GTP*γ*S (Cytoskeleton, cat. no. BS01) was added to HeLa cytosolic extracts, S100 [78] to give a 5 µM final GTP*γ*S concentration and incubated at room temperature for 15 min with gentle rotation. The reaction was stopped by transferring the tube to 4°C and adding 1/10th volume of STOP buffer (supplied) and was then used for the G-LISA assay.

#### 4.9.2. Rhoa activation by serum stimulation

Cells grown in DMED plus 10% fetal bovine serum were starved in serum-free medium for 24 h and incubated in the presence or absence of the compound at indicated concentrations for 30 min and subsequently stimulated with 10% FBS for 15 min. For G-LISA RhoA activity measurement, cells were harvested in lysis buffer (20 mM Tris-HCl, pH 7.6, 100 mM NaCl, 1% TritonX-100, 10 mM MgCl_2_, 2 mM NaF and a protease inhibitor cocktail). Lysates were normalized, equal amounts of protein were incubated for the assay.

### 4.10. Measurements of viability

Cells were plated in 384-well plates at a density of 800 cells per well in 22.5 µl of media. After 24 h, 2.5 µl drug diluted in media, was added to each well. Following 5 days of incubation, viability was assessed using CellTiter-Glo (Promega) according to the manufacture's protocol. Luminescence in each well was measured using a Synergy Neo2 plate reader (BioTek).

## Data Availability

The data are provided in the electronic supplementary material [79].
